# Association between prenatal exposure to perfluoroalkyl substances and asthma in 5-year-old children in the Odense Child Cohort

**DOI:** 10.1186/s12940-019-0541-z

**Published:** 2019-11-15

**Authors:** Iben Have Beck, Clara Amalie Gade Timmermann, Flemming Nielsen, Greet Schoeters, Camilla Jøhnk, Henriette Boye Kyhl, Arne Høst, Tina Kold Jensen

**Affiliations:** 1 Department of Environmental Medicine, Institute of Public Health, University of Southern Denmark, Odense, Denmark; 2 Environmental Risk and Health Unit, Flemish Institute for Technological Research (VITO), Mol, Belgium; 3 Department of Biomedical Sciences, University of Antwerp, 2000 Antwerp, Belgium; 4 Odense Patient data Explorative Network (OPEN), Odense, Denmark; 5 Hans Christian Andersen Children’s Hospital, Odense University Hospital, Odense, Denmark

**Keywords:** Perfluoroalkyl substances, PFAS, Prenatal exposure, Asthma, The Odense Child Cohort, Preschool, Children

## Abstract

**Background:**

Asthma is the most common non-communicable disease in children. Prenatal exposure to perfluoroalkyl substances (PFASs), a group of persistent environmental chemicals with endocrine disrupting abilities, has been associated with immunomodulation and may contribute to the aetiology of asthma. We investigated the associations between prenatal exposure to five PFASs and asthma in 5-year-old children.

**Methods:**

We studied 981 mother-child pairs within the Odense Child Cohort (OCC), Denmark. We measured perfluorooctane sulfonic acid (PFOS), perfluorooctanoic acid (PFOA), perfluorohexane sulfonic acid (PFHxS), perfluorononanoic acid (PFNA) and perfluorodecanoic acid (PFDA) in maternal serum donated in early pregnancy. A standardized questionnaire based on the International Study of Asthma and Allergies in Childhood (ISAAC) was used to assess wheeze, self-reported asthma and doctor-diagnosed asthma among children at age 5 years. Associations were examined using logistic regression analyses adjusting for parity, maternal educational level, maternal pre-pregnancy BMI, asthma predisposition and child sex.

**Results:**

Among the 5-year-old children 18.6% reported wheeze and 7.1% reported asthma. We found no association between prenatal exposure to PFAS and doctor-diagnosed asthma or wheeze. Prenatal PFAS exposure was associated with self-reported asthma, although only significant for PFNA (OR = 1.84, 95% CI 1.03,3.23).

**Conclusion:**

Our findings support the suggested immunomodulatory effects of PFASs, however, additional studies are warranted. In order to verify our findings, it is important to re-examine the children with postnatal measurements of serum PFAS concentrations and additional clinical diagnostic testing at an older age where an asthma diagnosis is more valid.

## Background

Asthma is the most common non-communicable disease in children [1]. The prevalence of asthma in Danish 5-year-old children is estimated to be 12% [2], it is, however, difficult to verify asthma among preschool children [3, 4]. Asthma diagnosis covers a variety of diseases with similar presentations [5] e.g. recurrent airway obstruction, inflammation of the airways and bronchial hyper-responsiveness to different stimuli [6].

Perfluoroalkyl substances (PFASs) are persistent chemicals with long elimination half-lives of 4 to 8 years in humans [7]. They are widely used in the industrial and commercial production of water-resistant fabrics, grease proof materials and non-stick coatings [8, 9]. Humans are exposed to these substances through ingestion of contaminated food and drinking water as well as inhalation of indoor air particles [10–12]. PFASs circulates through the placenta exposing the foetus [13, 14], and some PFASs are detectable in almost all human serum-samples [9, 15]. Perfluorooctane sulfonic acid (PFOS) and perfluorooctanoic acid (PFOA) are the oldest and most investigated PFASs [16].

Their adverse health effects have been documented and they have more or less been phased out from most industries, leading to declining serum concentrations [17, 18]. For other long-chain PFASs including perfluorohexane sulfonic acid (PFHxS), perfluorononanoic acid (PFNA), and perfluorodecanoic acid (PFDA) phase-out has not been adequately implemented and serum concentrations are increasing in some countries [19]. Previous epidemiological studies have reported associations between prenatal PFAS exposure and immune-related findings e.g. lower antibody responses to childhood immunizations in 5 and 7-year-old children [20]. Thus, PFASs have the ability to modulate the immune system, especially during vulnerable periods of foetal development [21] and could thereby contribute to the aetiology of asthma.

Few studies have investigated the association between prenatal PFASs exposure and asthma or asthma symptoms in children and findings are inconsistent [22–26]. None of the previous conducted studies reported significant associations between prenatal PFAS exposure and doctor-diagnosed asthma, while some found prenatal PFAS exposure to be associated with wheeze [22, 23]. Among these studies, population sizes vary from 99 to 1558 participants and children are between 1 and 10 years of age when assessed. Due to the sparse amount of research conducted in this field, dissimilarities in study settings may play a role in the differing findings.

We therefore seek to contribute and strengthen the evidence within this field of environmental science by investigating the association between prenatal exposure to five PFASs and wheeze, self-reported asthma and doctor-diagnosed asthma among 981, 5-year-old children in the Odense Child Cohort (OCC).

## Materials and methods

### Study population and setting

The OCC is an ongoing prospective cohort study. The cohort participants were recruited during the period 2010 to 2012 by inviting all newly pregnant women (*n* = 6707) residing in Odense Municipality to participate. Of the eligible women who met the inclusion criteria 2874 (43%) agreed to participate [27]. At the time of inclusion, a blood sample was drawn, and the participants filled out a questionnaire on their general health, lifestyle and social factors. Information on maternal educational level, smoking status during pregnancy, and pre-pregnancy body mass index, kg/m^2^ (BMI) was obtained from the questionnaire. Maternal educational level was categorized into 3 groups; lower (high school or less), intermediate (high school + 1–4 years of education) and higher (high school + more than 4 years of education). Information on maternal age, parity (nulliparous or multiparous), and child sex, birth weight and gestational age (GA) were derived from the birth record. Information regarding duration of breastfeeding was obtained from questionnaires completed at 3 and 18 months. During the study period the drop-out was minimal (4%) and there are currently 2549-registered mother-child pairs (March 2018).

### PFAS analysis

Maternal serum donated at inclusion (GA week 8–16) was analysed for PFAS concentrations in 1628 samples. Analyses included the following compounds; PFOS, PFOA, PFHxS, PFNA and PFDA. Initially a subset of 649 samples were analysed, of these, 200 were selected randomly [28], while the rest were selected based on availability of information from questionnaires, birth records and clinical three-months examinations of the child [29]. Finally, the remaining 979 samples were analysed in January 2019. Until analysis the samples were stored at − 80 °C. PFAS concentrations were estimated using on-line solid phase extraction followed by liquid chromatography and triple quadrupole mass spectrometry (LC-MS/MS) at the Department of Environmental Medicine, University of Southern Denmark. The initial analyses were performed between September 2011 and September 2013 and the remaining in 2019. The within-batch coefficients of variation (CVs) were < 3% and the between batch CVs for all sets analysed were < 10.5%. Quality control and quality assessment were based on certified reference material (NIST1958) from the National Institute of Standards and Technology [28]. The limit of quantification (LOQ) was 0.03 ng/ml for all compounds. PFOS, PFOA, PFNA and PFDA were detected in all samples in this study (LOQ > 0.03 ng/ml), while 0.5% (5/981) of the included women had a PFHxS concentration below the LOQ. These 5 were reported as LOQ/2.

### Assessment of asthma

A modified Danish version of the standardized questionnaire developed by the International Study of Asthma and Allergies in Childhood (ISAAC) [30, 31] completed by parents at child age 5 years, was used for asthma assessment. We selected three outcomes associated with asthma; wheeze, self-reported asthma and doctor-diagnosed asthma. Wheeze was assessed by the question “Has the child experienced whistling or wheezing (asthmatic) respiration at any time since the age of 3 years?”. If the parents confirmed, they were asked how many episodes of wheezing the child had experienced within the last 12 months. Self-reported asthma was defined as at least 3 episodes of wheezing (each lasting more than a day), during the past 12 months. Doctor-diagnosed asthma was defined by a positive answer to the question “Does the child have doctor-diagnosed asthma?”. Children whose parents indicated that the child had not experienced any wheezing episodes and did not answer the next questions regarding self-reported and doctor-diagnosed asthma, were assumed not to have asthma. When parents reported both doctor-diagnosed asthma and self-reported asthma in a child, the child was categorized only as doctor-diagnosed with asthma. Data concerning family history of asthma, concomitant dermatitis or rhinitis, smoking in the household and living with pet(s) were derived from the same questionnaire completed when the child was 5 years [31].

### Statistical analyses

Differences in serum PFAS concentrations among study participants and excluded participants and among the study participants with and without asthma or wheeze were assessed according to child, maternal and upbringing characteristics using Mann-Whitney and Kruskal-Wallis tests. Maternal, birth and child characteristics among mother-child pairs reporting to have or not have wheeze, self-reported asthma and doctor-diagnosed asthma were compared using Chi2-test and Mann-Whitney tests.

Logistic regression models were used to analyse the association between PFAS exposures and asthma. As PFASs distributions were positively skewed, all PFASs concentrations were converted using natural logarithm (ln) in order not to violate model assumptions, and estimates as well as 95% confidence intervals (CI) from the models were back transformed to express the odds ratios (ORs) of respiratory health outcomes (wheeze, self-reported asthma and doctor-diagnosed asthma) associated with a doubling of maternal PFASs. Potential confounders were identified through directed acyclic graphs (DAG) using Dagity software. Through the DAG we detected parity, maternal educational level, maternal pre-pregnancy BMI as confounders (Additional file 1). Furthermore, asthma predisposition and child sex are strong predictors of childhood asthma and they were therefore included in the models to reduce imprecision. In the final model we adjusted for parity, maternal educational level, maternal pre-pregnancy BMI, asthma predisposition and child sex. We also tested whether the associations were modified by sex by inserting interaction terms (PFAS*child sex) in the logistic regression models. Hosmer-Lemeshow goodness-of-fit test was used for testing the robustness of the logistic regression models.

Results with *p*-values< 0.05 were considered statistically significant. Statistical analyses were conducted using the statistical program STATA version 14.

## Results

**Fig. 1:**
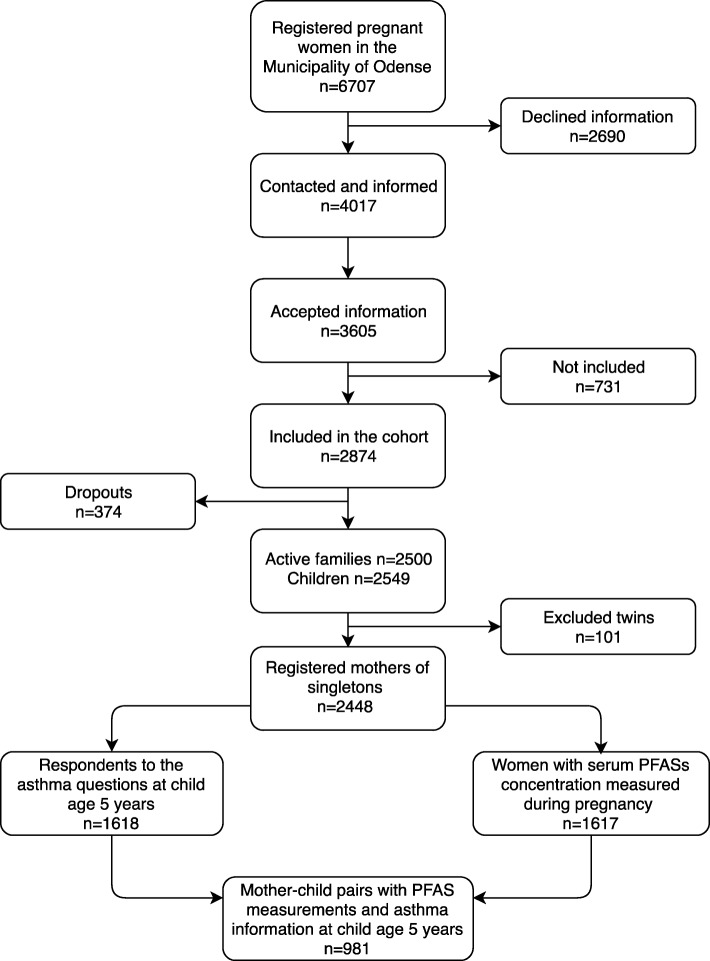
Flowchart presenting selection of study population. The 981 mother-child pairs derived from the Odense Child Cohort

A total of 2448 singleton mother-child pairs are currently active in the OCC. Of these 1618 responded to the ISAAC based questionnaire at child age 5 years and PFAS concentrations were analysed in 1617 maternal serum samples. A total of 981 mother-child pairs had answered both the ISAAC questionnaire and had serum PFASs measured (Fig. 1). Included participants reported longer duration of breastfeeding and had higher PFOS concentrations compared to the excluded women (median 7.73 ng/ml vs. 7.26 ng/ml) (data not shown). No differences between excluded and included participants in respiratory outcomes (wheeze, self-reported asthma or doctor-diagnosed asthma) or remaining covariates were found.

Of the 981 participating children, 52.1% were boys, the mean (SD) birth weight was 3533 (515) grams, and 3.8% were born preterm (< 37 weeks). The mean (SD) duration of breastfeeding was 32.5 (20.3) weeks, mean (SD) maternal age 30.9 (4.3) years, mean (SD) pre-pregnancy BMI was 24.3 (4.4) kg/m^2^, 57.6% of the women were nulliparous, 4% smoked during pregnancy and 23.1% had completed a higher education*.* From the age of 3 to 5 years, 18.6% of the children had experienced wheeze (*n* = 182), 7.1% had asthma (*n* = 69) of which 4.5% were doctor-diagnosed asthma (*n* = 44). Children with asthma or wheeze were more likely to have concomitant atopic dermatitis and to have a parent diagnosed with asthma. Their mothers tended to be younger, more overweight and with lower educational level (Table 1). Significantly more boys than girls had wheeze or doctor-diagnosed asthma and significantly more mothers had been smoking during pregnancy among children with doctor-diagnosed asthma. Children with self-reported asthma were breastfed for a shorter period and their mothers were more often nulliparous (Table 1).

**Table 1:** Distribution (%) of asthma related health outcomes in 5-year-old children (*n* = 981) according to child, maternal and upbringing characteristics in the Odense Child Cohort

			Wheeze		Self-reported asthma		Doctor-diagnosed asthma	
	n	%	Yes % 19.0 (*n* = 186)	No % 81.0 (*n* = 795)	Yes % 2.6 (*n* = 25)	No % 97.4 (*n* = 956)	Yes % 4.5 (*n* = 44)	No % 95.5 (*n* = 937)
Sex								
Boy	511	52.1	64.5 *	49.2 *	52.0	52.1	86.4 *	50.5 *
Girl	470	47.9	35.8 *	50.8 *	48.0	47.9	13.6 *	49.5 *
Birthweight (grams)								
< 2500	25	2.6	2.2	2.6	4.0	2.5	2.3	2.5
2500–4500	928	94.6	94.1	94.8	96.0	94.6	90.9	94.8
> 4500	28	2.8	3.7	2.6	0.0	2.9	6.8	2.7
Preterm (< 37 weeks)								
Yes	37	3.8	4.3	3.6	4.0	3.8	6.8	3.6
No	944	96.2	95.7	96.4	96.0	96.2	93.2	96.4
Breastfeeding^a^								
< 4 weeks	87	10.8	12.1	10.5	30.0 *	10.3 *	8.6	10.9
4–19 weeks	132	16.4	17.8	16.0	10.0 *	16.5 *	14.3	16.5
> 19 weeks	587	72.8	70.1	73.5	60.0 *	73.2 *	77.1	72.6
Age (years)								
< 28	250	25.5	31.2	24.1	36.0	25.2	34.1	25.1
28–34	492	50.2	46.2	51.1	52.0	50.1	47.7	50.3
> 34	239	24.3	22.6	24.8	12.0	24.7	18.2	24.6
BMI (kg/m2)								
< 20	109	11.1	11.3 *	11.1 *	8.0	11.2	11.4	11.1
20–25	543	55.4	46.2 *	57.5 *	52.0	55.4	45.5	55.8
> 25	329	33.5	42.5 *	31.4 *	40.0	33.4	43.2	33.1
Parity								
Nulliparous	565	57.6	56.5	58.0	68.0	57.3	50.0	58.0
Multiparous	416	42.4	43.5	42.0	32.0	42.7	50.0	42.0
Smoking								
Yes	39	4.0	5.4	3.7	4.0	4.0	11.4 *	3.6 *
No	942	96.0	94.6	96.3	96.0	96.0	88.6 *	96.4 *
Education level^b^								
Lower	244	25.2	33.7 *	23.2 *	32.0	25.0	32.6	24.8
Intermediate	502	51.7	44.8 *	53.4 *	40.0	52.0	53.5	51.7
Higher	224	23.1	21.5 *	23.4 *	28.0	23.0	13.9	23.5
Family asthma								
Yes	156	15.9	25.3 *	13.7 *	36.0 *	15.4 *	36.4 *	14.9 *
No	825	84.1	74.7 *	86.3 *	64.0 *	84.6 *	63.6 *	85.1 *
Doctor-diagnosed atopic dermatitis								
Yes	60	6.1	10.2 *	5.2 *	20.0 *	5.8 *	11.4	5.9
No	921	93.9	89.8 *	94.8 *	80.0 *	94.2 *	88.6	94.1
Doctor-diagnosed rhinitis								
Yes	20	2.0	4.3 *	1.5 *	0.0	2.1	9.1 *	1.7 *
No	961	98.0	95.7 *	98.5 *	100.0	97.9	90.9 *	98.3 *
Smoking in household								
Yes	141	14.4	17.2	13.7	12.0	14.4	18.2	14.2
No	840	85.6	82.8	86.3	88.0	85.6	81.8	85.8
Pets in household^c^								
Indoor	327	35.5	41.6	33.9	40.0	35.3	47.7	34.8
Outdoor	50	5.4	4.3	5.7	4.0	5.5	2.3	5.6
No	545	59.1	54.1	60.4	56.0	59.2	50.0	59.6

a) Missing (*n* = 175). b) Missing (*n* = 11). c) Missing (*n* = 59)

* *p* < 0.05 (Chi^2^ test)

Higher PFASs concentrations were found in younger, nulliparous women and among women with lower educational level and with lower BMI (Additional file 2). Serum PFAS concentrations differed very little in mothers of children with and without wheeze or doctor-diagnosed asthma, but mothers of children with self-reported asthma tended to have higher serum PFAS concentrations (significant for PFNA) (Table 2).

**Table 2:** Median maternal serum-PFAS concentrations (ng/ml) and (25th – 75th percentiles) according to respiratory health outcomes

Maternal serum-PFAS concentration (ng/ml) median (25th–75th percentile)		Wheeze		Self-reported Asthma		Doctor-diagnosed Asthma	
	All *n* = 981	Yes *n* = 182	No *n* = 799	Yes *n* = 25	No *n* = 956	Yes *n* = 44	No *n* = 937
PFOS	7.73 (5.68–10.44)	7.69 (5.85–10.86)	7.73 (5.66–10.39)	8.09 (6.90–10.86)	7.72 (5.67–10.43)	7.32 (5.39–9.01)	7.73 (5.70–10.45)
PFOA	1.68 (1.13–2.35)	1.63 (1.17–2.30)	1.69 (1.11–2.36)	1.83 (1.37–2.59)	1.68 (1.12–2.34)	1.55 (1.09–1.97)	1.69 (1.13–2.37)
PFHxS	0.36 (0.24–0.50)	0.36 (0.25–0.50)	0.36 (0.24–0.50)	0.37 (0.30–0.48)	0.36 (0.24–0.50)	0.34 (0.25–0.51)	0.36 (0.24–0.50)
PFNA	0.65 (0.49–0.86)	0.66 (0.51–0.86)	0.65 (0.49–0.86)	0.75* (0.58–0.96)	0.64* (0.49–0.86)	0.61 (0.44–0.77)	0.65 (0.49–0.87)
PFDA	0.29 (0.22–0.40)	0.30 (0.22–0.43)	0.29 (0.22–0.40)	0.35 (0.19–0.46)	0.29 (0.22–0.40)	0.29 (0.22–0.39)	0.29 (0.22–0.40)

* *p* < 0.05, using the Mann-Whitney test

In logistic regression analyses a doubling in PFASs exposure was associated with increased odds of self-reported asthma. After adjustment for parity, maternal educational level, maternal pre-pregnancy BMI, asthma predisposition and child sex, the association remained, although only statistically significant for PFNA (OR = 1.84, 95% CI 1.03,3.23). No associations between prenatal PFAS exposure and wheeze or doctor-diagnosed asthma were found (Table 3).

**Table 3:** Adjusted odds ratio (OR) and 95% confidence intervals (95% CI) for asthma outcomes (wheeze, self-reported and doctor-diagnosed asthma) by multiple logistic regression in the 5-year-old offspring for a doubling in maternal serum-PFAS concentrations (ng/ml). Results also stratified by sex

All (*n* = 981) Boys/ Girls (*n* = 511/470)	n	Wheeze		Self-reported asthma		Doctor-diagnosed asthma	
		n	OR (95% CI)	n	OR (95% CI)	n	OR (95% CI)
PFOS							
Unadjusted model	981	182	0.99 (0.78,1.26)	25	1.25 (0.68,2.32)	44	0.80 (0.53,1.23)
Adjusted model^a^	970	177	1.01 (0.79,1.30)	25	1.22 (0.65,2.28)	43	0.83 (0.52,1.31)
Boys^b^	507	117	1.02 (0.74,1.39)	13	2.39 (0.92,6.21)	37	0.74 (0.46,1.20)
Girls^b^	363	560	1.01 (0.67,1.52)	12	0.67 (0.29,1.53)	6	1.60 (0.46,5.59)
p^i^-value			0.96		0.048*		0.26
PFOA							
Unadjusted model	981	182	1.01 (0.83,1.23)	25	1.61 (0.99,2.63)	44	0.84 (0.58,1.22)
Adjusted model^a^	970	177	0.98 (0.78,1.23)	25	1.57 (0.93,2.68)	43	0.81 (0.53,1.22)
Boys^b^	507	117	0.94 (0.71,1.23)	13	2.17* (1.07,4.42)	37	0.72 (0.46,1.12)
Girls^b^	363	60	1.08 (0.75,1.55)	12	1.06 (0.49,2.30)	6	1.70 (0.63,4.56)
p^i^-value			0.52		0.17		0.11
PFHxS							
Unadjusted model	981	182	1.04 (0.87,1.25)	25	1.16 (0.74,1.84)	44	1.02 (0.73,1.43)
Adjusted model^a^	970	177	1.13 (0.93,1.38)	25	1.18 (0.73,1.90)	43	1.16 (0.78,1.71)
Boys^b^	507	117	1.03 (0.80,1.34)	13	1.33 (0.66,2.71)	37	0.89 (0.59,1.34)
Girls^b^	363	60	1.29 (0.94,1.77)	12	1.04 (0.55,1.98)	6	2.96* (1.26,6.96)
p^i^-value			0.28		0.61		0.013*
PFNA							
Unadjusted model	981	182	1.04 (0.81,1.34)	25	1.85* (1.06,3.23)	44	0.70 (0.43,1.13)
Adjusted model^a^	970	177	1.03 (0.79,1.33)	25	1.84* (1.03,3.28)	43	0.68 (0.41,1.14)
Boys^b^	507	117	0.93 (0.67,1.29)	13	2.11 (0.97,4.58)	37	0.58 (0.33,1.03)
Girls^b^	363	60	1.22 (0.79,1.87)	12	1.50 (0.64,3.49)	6	1.52 (0.47,4.97)
p^i^-value			0.32		0.56		0.15
PFDA							
Unadjusted model	981	182	1.16 (0.93,1.44)	25	1.37 (0.83,2.26)	44	0.93 (0.61,1.42)
Adjusted model^a^	970	177	1.16 (0.93,1.46)	25	1.44 (0.87,2.41)	43	0.93 (0.60,1.44)
Boys^b^	507	117	1.21 (0.92,1.60)	13	1.08 (0.51,2.29)	37	0.91 (0.57,1.45)
Girls^b^	363	60	1.08 (0.73,1.58)	12	1.88 (0.93,3.80)	6	1.11 (0.38,3.29)
p^i^-value			0.62		0.28		0.73

a: adjusted for parity, maternal educational level, maternal pre-pregnancy BMI, asthma predisposition and child sex

b: adjusted for parity, maternal educational level, maternal pre-pregnancy BMI and asthma predisposition

i: p-value for PFAS*child sex interaction

*: p < 0.05

Significant interactions between PFOS and sex were found in relation to self-reported asthma and between PFHxS and sex in relation to doctor-diagnosed asthma, thus, the analyses were stratified. After stratification, the only significant ORs were between PFHxS and doctor-diagnosed asthma in girls and between PFOA and self-reported asthma in boys. In the latter, the interaction was not statistically significant for a difference between sexes and there was no association in the combined (boys and girls) model. The association between prenatal exposure to PFAS and doctor-diagnosed asthma, tended to be stronger in girls, statistically significant for PFHxS (OR = 2.96 (95% CI 1.26,6.96) (Table 3). However only 6 girls had doctor-diagnosed asthma at age 5 years. Opposite tendencies were detected in associations between prenatal exposure to PFAS and self-reported asthma, where a doubling in PFOS, PFOA and PFNA exposure was associated with a doubling in risk of self-reported asthma in boys statistically significant in PFOA (OR = 2.17 (95% CI 1.07,4.42). The associations between prenatal exposure to PFAS and wheeze virtually remained unchanged after stratification by sex (Table 3).

## Discussion

In this prospective cohort study among 981 mother-child pairs, we found higher ORs for self-reported asthma among 5-year-old children prenatally exposed to PFAS although only statistically significant for PFNA exposure. After stratification by sex the associations between prenatal PFAS exposure and self-reported asthma were stronger in boys, whereas the association between PFAS exposure and doctor-diagnosed asthma were more pronounced in girls. However, only 6 girls had doctor-diagnosed asthma at age 5 years and only two interactions were significant. No significant association between prenatal exposure to PFASs and wheeze at age 5 years was found. The lack of association between prenatal PFAS exposure and wheeze may be due to the fact that young children often experience non-asthmatic wheeze related to common colds, bronchitis or upper respiratoy tract infections [4, 32], and wheeze may be too divergent a diagnosis to detect associations. Also, the children were examined at a young age, where the assessment of asthma is less valid [3]. However, children with doctor-diagnosed asthma as early as the age of 5 years may represent a more severe phenotype more likely to be inherited and therefore less associated to environmental exposures [33] than self-reported asthma. This may explain the lack of association among children with doctor-diagnosed asthma. Boys are more susceptible to childhood asthma than girls [33, 34], and it has been suggested that girls are more susceptible to the immunomodulatory effects of prenatal PFAS exposure [35, 36], which is consistent with our stratified findings among children with doctor-diagnosed asthma, but not among children with self-reported asthma. The reduced number of cases in each group after stratification yield for cautious interpretation of these findings.

We cannot rule out that our finding have occurred by chance, as no other studies to our knowledge have found significant associations between prenatal PFNA exposure and asthma related outcomes. None of the studies have investigated self-reported asthma as a single health outcome, however, several studies have documented associations between prenatal PFNA exposure and outcomes with infectious origin [25, 37] and antibody responsiveness [25] in 0 to 3-year-old children, indicating that PFNA may have immunomodulatory abilities.

Results from comparable studies are conflicting. A study among 1558 Japanese mother-child pairs from the Hokkaido Cohort found a significant association between prenatal PFHxS exposure and wheeze among 4-year-old children, nonetheless, were found non-significant after covariate adjustment [22]. In the INUENDO cohort, a significant inverse association between prenatal PFOA exposure and wheeze among 5- to 9-year old Ukrainian children was found, whereas no such inverse association was found among the Greenlandic children, who were exposed to PFOA concentrations twice as high as the Ukrainian children [23] (Additional file 3). The OCC children were exposed to lower concentrations of PFAS than children in these cohorts (Additional file 3) which may explain the lack of association in our study. Three other cohort studies [22, 25, 26] with PFAS exposure levels comparable to women in our study found no association between prenatal exposure to PFASs and wheeze, which is consistent with our findings. In addition, we did not find any significant association between prenatal PFAS exposure and doctor-diagnosed asthma, which is consistent with findings from 4 different studies conducted among 1- to 3-year-olds [25] and 2- and 10-year-olds [26] from Norway, 5- to 9-year-olds from Greenland and the Ukraine [23] and among 5-year-olds from the Faroe Islands [24] (Additional file 3). None of these 4 studies conducted sex stratified analyses which hinder comparison of our findings indicating sex differences.

Women in this study had lower serum-PFAS concentrations than women from Japan, Greenland and the Faroe Islands [22–24] but higher than women from Norway and the Ukraine [23, 25] (Additional file 3). These differences are consistent with varying PFAS exposure through dietary intake e.g. residents of Japan, Greenland and the Faroe Islands are known to eat more seafood and marine mammals, which are known sources of PFAS exposure [10, 38, 39]. In addition, time trends in PFAS exposure may explain the observed differences. Other discrepancies among study populations, such as geographic areas of residence and diverging definitions of asthma related outcome variables, sizes of study populations and child age, may affect the observed associations in published studies (Additional file 3).

Asthma is characterized by hyper responsiveness to allergens caused by allergen-specific IgE and a shift in the T-helper (Th)-1/Th-2 balance towards Th-2 [21, 40]. Animal models are indicative of immunomodulatory effects after PFASs exposure [41, 42]. Studies in mice report that increasing exposure to PFOS, creates a shift in the cytokine balance toward Th-2, leading to suppression of the cellular response and enhancement of the humoral response [43, 44]. A shift in the cytokine profiles towards Th2, will favour an allergic response [21, 45], and prenatal exposure to PFAS may thus contribute in the aetiology of asthma. Toxicological studies provide evidence that exposure to PFOA and PFOS increase the risk for immunomodulation [46]. Studies in mice report associations between PFOS and PFOA exposure and immunomodulatory effects with both prenatal- [47] and concurrent PFAS exposure [41]. Studies investigating concurrent exposure are not directly comparable to studies of prenatal exposure, however, the adverse effects could hypothetically be particular harmful if exposure occur when the immune system is developing in utero. In epidemiological studies, high prenatal exposure to PFASs have been associated with lower antibody responses to childhood immunizations in children aged 3 years [25], 5 and 7 years [20]. Prenatal PFAS exposure have also been associated to childhood infections [25, 26, 48], supporting the growing evidence of immunomodulatory effects of prenatal exposure to PFAS.

This study has several strengths. It is a large, population-based, prospective study which secures temporality. Furthermore, we have assessed asthma through international standardized and validated questionnaires [30]. As a measure of prenatal PFASs exposure, we used maternal serum PFAS concentrations collected at GA week 8–16. PFASs can circulate through the placenta and PFAS concentrations in maternal and cord blood are verified highly correlated [13, 14]. Maternal serum concentrations of PFAS are known to decrease throughout pregnancy [17], however, the long half-lives of PFASs reduce the likelihood of large fluctuations in PFASs concentrations during pregnancy [49]. PFASs concentrations measured in early pregnancy are therefore considered reliable to the exposure level during the entire pregnancy.

Women participating in the OCC were older, more often nulliparous and fewer were smokers compared to the background population [27]. This may partially explain the relative low asthma prevalence (7.1%) in our study, as both young maternal age and smoking during pregnancy are well known risk factors for asthma in the offspring [50, 51]. A Danish study of more than 900,000 Danish 5-year-old children born from 1997 to 2011, reported a 12% asthma prevalence at age 5 years. The asthma diagnosis was based on hospital contacts and disease-specific dispensed prescribed medications [2], which may overestimate the prevalence. As our population may be healthier than the general population, we cannot rule out the possibility of selection bias, however, we compared women across PFAS exposure, so whether they represented the general population is therefore less important. In addition, participants had no knowledge of their PFAS exposures or asthma symptoms in their child at enrolment. It is therefore unlikely to have affected their participation.

Assessment of asthma in preschool children is challenging and is often based on symptoms and response to medication [3]. To verify the asthma diagnosis diagnostic tests such as spirometry has to be carried out [3, 4]. These test are not suitable for preschool children, and the diagnosis is therefore less valid for younger children [3]. The worldwide epidemiological research program ISAAC has developed an international, standardized questionnaire [52] by which self-reported asthma can be assessed [30]. Some parents may have misinterpreted recurrent colds or respiratory infection symptoms as asthma [32], and misclassification is therefore likely. However, parents were unaware of their PFAS exposure when responding to the questionnaire, and misclassification is therefore most likely non-differential leading to underestimation of the associations.

We investigated a wide range of potential confounders without notable changes in the estimates, but unmeasured confounding may be present. Recent studies have found that increasing maternal serum-PFAS concentrations reduces the duration of breastfeeding [53, 54]. Breastfeeding is a source of postnatal PFAS exposure for infants [11, 55] but has also been associated with reduced risk of childhood asthma [56]. We did not adjust for duration of breastfeeding, as we regarded it to be a mediator (Additional file 1) [57]. A strong association between PFAS exposure and asthma among measles, mumps and rubella (MMR)-unvaccinated children was found in a Faroe Island cohort [24], but in the present study only 4 children were known not to be MMR vaccinated, which was not sufficient to test the hypothesis. Previous studies have found childhood serum-PFAS concentrations to be associated with childhood asthma [58–60], therefore, as the immune system is developed and matured both pre- and postnatally [40], it will be of relevance to measure serum-PFAS concentrations in the children.

In conclusion, our findings suggest an association between prenatal exposure to PFASs and self-reported asthma in the offspring. We found no significant associations between prenatal exposure to PFASs and doctor-diagnosed asthma or wheeze, and additional studies are warranted. Our findings support the reported immunomodulatory effects of PFASs, and are a valuable contribution to research conducted within the public health field. In future studies, it is recommended to analyse the children’s serum PFAS concentrations and to re-examine them with e.g. spirometry to validate the asthma diagnosis at a later age.

## Supplementary information

**Additional file 1: Figure S1.** The causal network between prenatal PFAS levels and asthma, presented in a directed acyclic graph (DAG).

**Additional file 2: Table S1.** Maternal PFAS concentrations (ng/mL), median and 25–75 percentiles according to maternal and child characteristics in 981 mother-child pairs in Odense Child Cohort.

**Additional file 3: Table S2.** Overview of epidemiological cohort studies, investigating associations between prenatal PFASs exposure and respiratory health outcomes in children, Identified through structured PubMed search

## Data Availability

The data set used and analysed during the current study are available from the corresponding author on reasonable request.

## Abbreviations

°C: degrees Celsius
BMI: Body mass index (kg/m^2^)
CI: Confidence interval
CV: Coefficients of variation
DAG: Directed acyclic graphs
GA: Gestational age
IgE: Immunoglobulin E
ISAAC: International Study of Asthma and Allergies in Childhood
LC-MS/MS: liquid chromatography and triple quadrupole mass spectrometry
ln: Natural logarithm
LOQ: Limit of quantification
ml: millilitre
MMR: measles, mumps and rubella
ng: Nano gram
OCC: Odense Child Cohort
OPEN: Odense Patient data Explorative Network
OR: Odds ratio
PFAS: Perfluoroalkyl substance
PFDA: Perfluorodecanoic acid
PFHxS: Perfluorohexane sulfonic acid
PFNA: Perfluorononanoic acid
PFOA: Perfluorooctanoic acid
PFOS: Perfluorooctane sulfonic acid
SD: Standard deviation
Th: T-helper
