# Supplementary material for: Association between prenatal exposure to perfluoroalkyl substances and asthma in 5-year-old children in the Odense Child Cohort
Source: Environ Health. 2019 Nov 15;18:97. doi: 10.1186/s12940-019-0541-z (PMC6858758; doi:10.1186/s12940-019-0541-z)
Supplement: Supplementary file 1 — Additional file 1: Figure S1. The causal network between prenatal PFAS levels and asthma, presented in a directed acyclic graph (DAG). [file 12940_2019_541_MOESM1_ESM.docx]

Additional file 1

The causal network between prenatal PFAS levels and asthma, presented in a directed acyclic graph (DAG).

Additional Figure 1:

The causal network between prenatal PFASs levels and asthma after adjustment for parity, maternal educational level, maternal pre-pregnancy BMI, asthma predisposition and child sex, presented in a DAG.


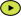
 exposure,
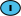
 outcome,
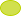
 ancestor of exposure,
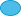
 ancestor of outcome,
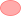
 ancestor of exposure and outcome,


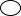
 adjusted variable,
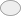
 unobserved variable,
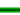
 causal path
