# Supplementary material for: Association between prenatal exposure to perfluoroalkyl substances and asthma in 5-year-old children in the Odense Child Cohort
Source: Environ Health. 2019 Nov 15;18:97. doi: 10.1186/s12940-019-0541-z (PMC6858758; doi:10.1186/s12940-019-0541-z)
Supplement: Supplementary file 2 — Additional file 2: Table S1. Maternal PFAS concentrations (ng/mL), median and 25–75 percentiles according to maternal and child characteristics in 981 mother-child pairs in Odense Child Cohort. [file 12940_2019_541_MOESM2_ESM.docx]

Additional file 2

Additional Table 1. Maternal PFAS concentrations (ng/mL), median and 25-75 percentiles according to maternal and child characteristics in 981 mother-child pairs in Odense Child Cohort.

| Median  (percentiles) | % (n) | **PFOS**  (25%-75%) | **PFOA**  (25%-75%) | **PFHxS**  (25%-75%) | **PFNA**  (25%-75%) | **PFDA**  (25%-75%) |
| --- | --- | --- | --- | --- | --- | --- |
| Study population | 100 (981) | 7.73 (5.68-10.44) | 1.68 (1.13-2.35) | 0.36 (0.24-0.50) | 0.65 (0.49-0.86) | 0.29 (0.22-0.40) |
| **Child characteristics** | | | | | | |
| Sex  Boy  Girl | 52.1 (511)  47.9 (470) | 7.84 (5.57-10.59)  7.54 (5.78-10.35) | 1.72 (1.16-2.41)  1.65 (1.10-2.29) | 0.36 (0.25-0.50)  0.34 (0.24-0.50) | 0.67 (0.49-0.86)  0.64 (0.48-0.86) | 0.29 (0.22-0.40)  0.29 (0.22-0.40) |
| Birthweight (grams) | |  | | | | |
| <2500  2500-4500  >4500 | 2.6 (25)  94.6 (928)  2.8 (28) | 7.73 (6.30-9.79)  7.74 (5.69-10.55)  6.86 (5.42-8.67) | 1.80* (1.30-2.50)  1.69* (1.13-2.36)  1.30* (0.93-1.77) | 0.38 (0.26-0.54)  0.36 (0.25-0.50)  0.34 (0.22-0.45) | 0.58 (0.47-0.84)  0.65 (0.49-0.87)  0.63 (0.45-0.80) | 0.27 (0.21-0.39)  0.29 (0.22-0.40)  0.27 (0.21-0.32) |
| Preterm (<37 weeks) | |  | | | | |
| Yes  No | 3.8 (37)  96.2 (944) | 7.73 (5.85-11.31)  7.72 (5.68-10.43) | 1.81 (1.24-2.60)  1.67 (1.12-2.34) | 0.44 (0.23-0.54)  0.36 (0.24-0.50) | 0.64 (0.45-0.89)  0.65 (0.49-0.86) | 0.27 (0.21-0.39)  0.29 (0.22-0.40) |
| Breastfeeding^a^  < 4 weeks  4-19 weeks  > 19 weeks | 10.8 (87)  16.4 (132)  72.8 (587) | 8.37 (6.05-10.42)  7.78 (6.22-11.59)  7.69 (5.52-10.42) | 1.76* (1.15-2.59)  1.96* (1.37-2.59)  1.63* (1.07-2.29) | 0.35 (0.25-0.48)  0.37 (0.27-0.53)  0.34 (0.23-0.49) | 0.67 (0.46-0.83)  0.67 (0.49-0.92)  0.64 (0.49-0.86) | 0.26 (0.20-0.39)  0.29 (0.22-0.42)  0.29 (0.22-0.40) |
| **Maternal characteristics during pregnancy** | | | | | | |
| Age (years)  <28  28-34  >34 | 25.5 (250)  50.2 (492)  24.3 (239) | 7.79* (5.87-10.)  8.11 *(6.13-10.62)  6.92* (5.06-9.74) | 1.89* (1.27-2.53)  1.73* (1.16-2.36)  1.34* (0.97-1.99) | 0.35 (0.23-0.47)  0.37 (0.25-0.52)  0.33 (0.24-0.49) | 0.69* (0.48-0.89)  0.66* (0.51-0.88)  0.60* (0.45-0.80) | 0.29* (0.23-0.40)  0.30* (0.23-0.41)  0.27* (0.20-0.39) |
| BMI (kg/m2)  <20  20-25  >25 | 11.1 (109)  55.4 (543)  33.5 (329) | 8.49 (6.19-10.87)  7.76 (5.74-10.67)  7.43 (5.55-9.80) | 1.68 (1.12-2.29)  1.74 (1.13-2.45)  1.58 (1.12-2.26) | 0.36* (0.26-0.52)  0.37* (0.25-0.52)  0.32* (0.23-0.45) | 0.67* (0.50-0.87)  0.68* (0.51-0.89)  0.61* (0.47-0.80) | 0.33* (0.24-0.44)  0.30* (0.23-0.42)  0.27* (0.20-0.35) |
| Parity  Nulliparous  Multiparous | 57.6 (566)  42.4 (415) | 8.50* (6.45-11.22)  6.89* (5.16-9.16) | 2.02* (1.56-2.69)  1.22* (0.84-1.72) | 0.40* (0.28-0.55)  0.30* (0.21-0.41) | 0.71* (0.53-0.95)  0.58* (0.44-0.75) | 0.30* (0.23-0.42)  0.27* (0.21-0.37) |
| Smoking  Yes  No | 4.0 (39)  96.0 (942) | 7.47 (5.54-8.52)  7.73 (5.68-10.57) | 2.08 (1.25-2.30)  1.67 (1.12-2.35) | 0.34 (0.26-0.51)  0.36 (0.24-0.50) | 0.61 (0.47-0.89)  0.65 (0.49-0.86) | 0.24 (0.19-0.40)  0.29 (0.22-0.40) |
| Educational Level^b^  Lower  Intermediate  Higher | 25.2 (244)  51.7 (502)  23.1 (224) | 7.89 (5.91-11.00)  7.85 (5.92-10.29)  7.41 (5.29-10.44) | 1.83* (1.24-2.49)  1.65* (1.10-2.33)  1.62* (1.05-2.30) | 0.34* (0.24-0.47)  0.35* (0.25-0.50)  0.39* (0.26-0.56) | 0.66 (0.49-0.89)  0.64 (0.49-0.83)  0.66 (0.50-0.90) | 0.29 (0.22-0.39)  0.28 (0.22-0.39)  0.31 (0.22-0.43) |
| *: p<0.05 using Mann-Whitney test for characteristics with 2 parameters, Kruskal-Wallis test for characteristics with more than 2 parameters.  a) Missing (n=175).  b) Missing (n=11) | | | | | | |
