# Supplementary material for: Association between prenatal exposure to perfluoroalkyl substances and asthma in 5-year-old children in the Odense Child Cohort
Source: Environ Health. 2019 Nov 15;18:97. doi: 10.1186/s12940-019-0541-z (PMC6858758; doi:10.1186/s12940-019-0541-z)
Supplement: Supplementary file 3 — Additional file 3: Table S2. Overview of epidemiological cohort studies, investigating associations between prenatal PFASs exposure and respiratory health outcomes in children, Identified through structured PubMed search [file 12940_2019_541_MOESM3_ESM.docx]

Additional file 3

Additional Table 2 Overview of epidemiological cohort studies, investigating associations between prenatal PFASs exposure and respiratory health outcomes in children, Identified through structured PubMed search

| **Title** | **Study population** | **Exposure; PFAS assessment** | **Outcome; asthma assessment** | **Findings** |
| --- | --- | --- | --- | --- |
| Prenatal exposure to perfluoroalkyl substances may be associated with altered vaccine antibody levels and immune-related health outcomes in early childhood [1]. | MoBa cohort, Norway.  99 mother-child pairs. | Sample collection: 2007- 2011  Sample type: Maternal plasma  Sample obtained: Time of delivery.  Analysed PFASs (medians ng/ml)  PFOS (5.50)  PFOA (1.10)  PFHxS (0.30)  PFNA (0.30) | Asthma- and allergy-related health outcomes in children up to the age of 3 years.  Q: ‘has your child been diagnosed with asthma by a doctor?’  ‘has your child had periods of more than ten days of dry cough, chest tightness or wheeze within the past 12 months?’  (Anti-vaccine antibody levels, common infectious diseases). | - No association found between PFAS concentrations and the allergy- and asthma-related health outcomes investigated. |
| Prenatal exposure to environmental chemical contaminants and asthma and eczema in school-age children [2]. | INUENDO birth cohort, Ukraine and Greenland.  1024 mother-child pairs.  Ukraine (n=492)  Greenland (n=532) | Sample collection: 2002-2004  Sample type: Maternal Serum  Sample obtained: During pregnancy (exact time not reported).  Analysed PFASs (medians ng/ml)  Ukraine Greenland  PFOS (4.88) PFOS (20.60)  PFOA (0.97) PFOA (1.79)  PFHxS (1.53) PFHxS (2.14)  PFNA (0.62) PFNA (0.73)  PFDA (0.16) PFDA (0.42) | Parents were interviewed, using ISAAC based questionnaires assessing:  Ever/current wheeze  Ever asthma.  Child age 5 to 9 years. | - In the Ukrainian sub-cohort PFOA was inversely associated with current wheeze, OR 0.64.  Adjusted for: Maternal allergy, smoking during pregnancy, educational level, maternal age, child sex, child age at follow-up, GA at blood sampling, parity, breastfeeding and birthweight. |
| Effects of prenatal exposure to perfluoroalkyl acids on prevalence of allergic diseases among 4-year-old children [3]. | Hokkaido Study on Environment and Children's health, Japan.  1558 mother-child pairs. | Sample collection: 2003-2009  Sample type: Maternal plasma  Sample obtained: GA week 28-32  Analysed PFASs (medians ng/ml)  PFOS (4.93); PFOA (2.01)  PFHxS (0.30); PFNA (1.18)  PFDA (0.52) | ISAAC phase three based questionnaires at child age 4 years.  Q: ‘Has your child had wheezing or whistling in the chest in the past 12 months?’ | - PFHxS was significantly associated with the prevalence of wheezing in the crude model, however no significant associations were found after relevant adjustment.  Adjusted for: Maternal age, maternal educational level, parental allergic history, number of older siblings, breast feeding, day care attendance, and ETS exposure. |
| Association between perfluoroalkyl substance exposure and asthma and allergic disease in children as modified by MMR vaccination [4]. | CHEF cohort, The Faroe Islands.  559 mother-child pairs. | Sample collection: 1997-2000  Sample type: Maternal serum  Sample obtained: GA week 34-36  Analysed PFASs (medians ng/ml)  PFOS (27.40)  PFOA (3.30)  PFHxS (4.40)  PFNA (0.60)  PFDA (0.30) | Asthma/allergy assessment through ISAAC based questionnaires at age 5 and 13.  Q: ‘has your child been diagnosed with or been suspected to suffer from asthma?  (Serum IgE measured in cord blood and serum at age 7. Skin prick test at age 13). | - Prenatal PFAS exposure was not associated with childhood asthma or allergic diseases regardless of MMR vaccination status.  Adjusted for: Parity, family history of eczema in children, allergic eczema and hay fever, maternal pre-pregnancy BMI, maternal smoking during pregnancy, maternal fish intake during pregnancy and duration on breastfeeding |
| Prenatal exposure to perfluoroalkyl substances (PFASs) associated with respiratory tract infections but not allergy- and asthma-related health outcomes in childhood [5]. | Environment and Childhood Asthma (ECA) Study, Norway.  641 mother-child pairs. | Sample collection: 1992-1993  Sample type: Cord blood  Sample obtained: At birth  Analysed PFASs (medians ng/ml)  PFOS (5.6)  PFOA (1.8)  PFHxS (0.3)  PFNA (0.2) | Doctor-diagnosed asthma at 10 years  Doctor-diagnosed wheeze (0-3, 3-10, 0-10 years)  Severity of obstructive airways disease by 2 years | -Prenatal PFAS exposure was not associated with asthma or wheeze.  Adjusted for: Sex |

1. Granum B, Haug LS, Namork E, Stolevik SB, Thomsen C, Aaberge IS, van Loveren H, Lovik M, Nygaard UC: **Pre-natal exposure to perfluoroalkyl substances may be associated with altered vaccine antibody levels and immune-related health outcomes in early childhood**. *J Immunotoxicol* 2013, **10**(4):373-379.

2. Smit LA, Lenters V, Hoyer BB, Lindh CH, Pedersen HS, Liermontova I, Jonsson BA, Piersma AH, Bonde JP, Toft G *et al*: **Prenatal exposure to environmental chemical contaminants and asthma and eczema in school-age children**. *Allergy* 2015, **70**(6):653-660.

3. Goudarzi H, Miyashita C, Okada E, Kashino I, Kobayashi S, Chen CJ, Ito S, Araki A, Matsuura H, Ito YM *et al*: **Effects of prenatal exposure to perfluoroalkyl acids on prevalence ofallergic diseases among 4-year-old children**. *Environment international* 2016, **94**:124-132.

4. Timmermann CA, Budtz-Jorgensen E, Jensen TK, Osuna CE, Petersen MS, Steuerwald U, Nielsen F, Poulsen LK, Weihe P, Grandjean P: **Association between perfluoroalkyl substance exposure and asthma and allergic disease in children as modified by MMR vaccination**. *J Immunotoxicol* 2017a, **14**(1):39-49.

5. Impinen A, Nygaard UC, Lodrup Carlsen KC, Mowinckel P, Carlsen KH, Haug LS, Granum B: **Prenatal exposure to perfluoralkyl substances (PFASs) associated with respiratory tract infections but not allergy- and asthma-related health outcomes in childhood**. *Environ Res* 2018, **160**:518-523.
